# Design and Implementation of an Eyewear-Integrated Infrared Eye-Tracking System

**DOI:** 10.3390/s26072065

**Published:** 2026-03-26

**Authors:** Carlo Pezzoli, Marco Brando Mario Paracchini, Daniele Maria Crafa, Marco Carminati, Luca Merigo, Tommaso Ongarello, Marco Marcon

**Affiliations:** 1 Dipartimento di Elettronica, Informazione e Bioingegneria, Politecnico di Milano, 20133 Milano, Italy; carlo.pezzoli@polimi.it (C.P.); marcobrando.paracchini@polimi.it (M.B.M.P.); danielemaria.crafa@polimi.it (D.M.C.); marco1.carminati@polimi.it (M.C.); 2 EssilorLuxottica Smart Eyewear Lab, EssilorLuxottica, 20121 Milano, Italy; luca.merigo@luxottica.com (L.M.); tommaso.ongarello@luxottica.com (T.O.)

**Keywords:** gaze tracking, assistive technologies, computer vision, eyes, position measurement, user interfaces

## Abstract

Eye-tracking is a key enabling technology for smart eyewear, supporting hands-free interaction, accessibility, and context-aware human–machine interfaces under strict constraints on size, power consumption, and computational complexity. While camera-based solutions provide high accuracy, their integration into lightweight and low-power wearable platforms remains challenging. This paper is a feasibility study for the design, simulation, and experimental evaluation of a photosensor oculography (PSOG) eye-tracking system that is fully integrated into an eyewear frame, based on near-infrared (NIR) emitters and photodiodes. The proposed approach combines simulation-driven optimization of the optical constellation, a multi-frequency modulation and demodulation scheme enabling parallel source discrimination and robust ambient-light rejection, and a resource-efficient signal acquisition pipeline suitable for embedded implementation. Eye rotations in azimuth and elevation are inferred from differential reflectance patterns of ocular regions (sclera, iris, and pupil) using lightweight regression techniques, including shallow neural networks and Gaussian process regression, selected to balance estimation accuracy with computational and power constraints. System performance is evaluated using a controllable artificial-eye platform under defined geometric and illumination conditions, enabling repeatable assessment of gaze-estimation accuracy and algorithmic behavior. Sub-degree errors are achieved in this controlled setting, demonstrating the feasibility and potential effectiveness of the proposed architecture. Practical considerations for translation to real-world smart eyewear, including human-subject validation, anatomical variability, calibration strategies, and embedded deployment, are discussed and identified as directions for future work. By detailing the optical design methodology, modulation strategy, and algorithmic trade-offs, this work clarifies the distinct contributions of the proposed PSOG system relative to existing frame-integrated and camera-free eye-tracking approaches, and provides a foundation for further development toward wearable and augmented-reality applications.

## 1. Introduction

Eye-tracking (ET) is a relevant and active research topic in both academic and industrial contexts due to its wide range of applications and scientific implications [1,2]. Understanding where and how individuals direct their gaze provides fundamental insights into human behavior, cognition, and decision-making processes, supporting research in psychology, neuroscience, and education, where eye-tracking studies contribute to the analysis of attention, perception, and learning mechanisms.

In applied settings, eye-tracking is increasingly used to improve human–machine interaction (HMI) and user interfaces. By identifying visual attention patterns, eye-tracking data can be leveraged to enhance the usability of websites, applications, and physical products, as well as to support market research and advertising through objective measurements of user engagement. Beyond usability, eye-tracking has significant potential in healthcare and assistive technologies, where eye-movement analysis supports the diagnosis of visual and neurological disorders and enables accessible interaction paradigms for individuals with motor or speech impairments.

Recent advances in portable and non-invasive eye-tracking technologies have expanded their applicability beyond laboratory environments toward wearable platforms, particularly smart glasses. In this context, eye-tracking enables hands-free and intuitive interaction by allowing systems to infer user intent directly from gaze behavior, complementing or replacing traditional input modalities such as touch, voice, or gestures. However, the integration of eye-tracking systems into eyewear introduces stringent constraints in terms of form factor, power consumption, computational resources, and user comfort, which remain key challenges for practical deployment.

In parallel, gaze estimation and pupil detection have also emerged as enabling technologies for broader applications beyond interaction, including soft biometrics and unsupervised eye-feature localization. For instance, Cazzato et al. [3] demonstrated the effectiveness of low-cost and calibration-free gaze estimators for soft-biometric identification, while Leo et al. [4] proposed a robust unsupervised approach to pupil localization in near-frontal images. Earlier work by D’Orazio et al. [5] explored eye-detection strategies for driver vigilance monitoring, highlighting the relevance of eye-related features in safety-critical scenarios.

The approach presented in this work is based on photosensor oculography (PSOG), a class of techniques that estimate eye rotations by analyzing the intensity of light reflected from different ocular regions. Early PSOG systems can be traced back to the pioneering work of Török [6], in which a light spot partially overlapping the sclera and iris was used to detect eye movements via changes in reflected intensity. Russo [7] later introduced an infrared-based configuration employing multiple photodiodes to estimate eye rotation from limbus displacement, achieving an accuracy of approximately 0.5° under constrained conditions. To improve signal-to-noise ratio, modulation and demodulation strategies between emitters and detectors were subsequently introduced [8].

More recently, PSOG has attracted renewed interest as a viable solution for frame-integrated eye-tracking, particularly in smart glasses and lightweight wearable devices with limited computational capacity. Representative examples include the works of Pezzoli et al. [9], Pettenella et al. [10], Li et al. [11], while the survey by Rigas et al. [12] provides a comprehensive overview of PSOG-based approaches. Owing to the simplicity of the sensing principle, PSOG systems can achieve high sampling rates with low-power electronics, making them attractive alternatives to camera-based solutions for resource-constrained platforms.

Hybrid approaches combining PSOG with video-oculography (VOG) have also been proposed [13,14], leveraging near-eye cameras to improve accuracy and enable a detailed analysis of rapid eye movements such as saccades and microsaccades. While these systems offer enhanced performance, they introduce additional complexity in terms of hardware, weight, power consumption, and processing requirements, which can limit their suitability for compact and lightweight eyewear [15].

The literature indicates that accurate eye-tracking, particularly for the analysis of fast eye dynamics, benefits from high-frequency sampling. Angelopoulos et al. [13] report acquisition rates on the order of 10 kHz as desirable for capturing microsaccades and ocular tremor, given that eye movements can reach velocities of approximately 300°/s and angular accelerations up to 24,000°/s^2^. While such rates represent challenging design targets for wearable systems, they motivate the development of sensing architectures capable of high-speed acquisition while maintaining low power consumption. Moreover, the combined analysis of saccades, microsaccades, and pupil dynamics has been shown to provide valuable indicators of cognitive load and stress [16,17], as well as the blink [18].

Motivated by these considerations, this work investigates a fully frame-integrated PSOG eye-tracking architecture designed to operate under wearable constraints. The proposed system employs four near-infrared illuminators and four photodiodes embedded in the half-frame of a pair of smart glasses. A parallel multi-frequency modulation and demodulation scheme is adopted to discriminate the contributions of individual emitters, maximize signal integration time, and improve robustness to ambient infrared interference, while reducing the processing burden on the acquisition electronics. Gaze estimation in azimuth and elevation is performed using lightweight regression models selected with embedded deployment in mind.

The system is evaluated using a controllable artificial-eye platform under defined geometric and illumination conditions, enabling repeatable assessments of feasibility and performance. While human-subject validation is beyond the scope of the present feasibility study, the proposed design establishes a foundation for future work addressing anatomical variability, calibration strategies, and on-device implementation in smart eyewear applications.

## 2. Materials and Methods

The operating principle of the proposed system relies on multiple infrared light sources positioned near the eye on the eyeglass frame. These sources emit modulated IR light directly towards the ocular surface. A series of infrared detectors, also mounted on the frame, are used to capture the light reflected from the ocular surface and surrounding areas.

The various parts of the eye’s visible surface (sclera, cornea, iris, pupil, eyelid, etc.) absorb, reflect, and diffuse the incident light in different ways. Therefore, the amount of IR light reflected from each part of the eye in various directions depends on the characteristics of the ocular surface and the angle of incidence of the light rays from the sources [6].

As the eye rotates in yaw and pitch, the ocular surfaces illuminated by the light sources change, resulting in corresponding changes in the amount of light received by each detector.

### 2.1. Simulations

To assess the behavior of the human eye when exposed to infrared light from the sources and to evaluate the reflections generated and recorded by the detectors, simulations were conducted using Ansys^®^ Zemax^®^ OpticStudio^®^ 2023 R1 (Ansys Inc., Canonsburg, PA, USA), hereafter referred to as OpticStudio. These simulations were also instrumental in determining the optimal positioning and tilting of both the light sources and detectors, as well as the emission angle of the light, and estimating the amount of optical power reaching the eye to ensure compliance with eye safety standards in the final prototype.

OpticStudio [19,20] is a software widely used in the optics field that allows users to model and simulate optical systems. It can be used for the design and analysis of lenses, cameras, microscopes, telescopes, and other optical systems. It can also model and simulate the behavior of the human eye [21], as it is a biological optical system. In the OpticStudio editor, users can define all the components of the system they wish to simulate by specifying the shape, material, and fine-tuning of the relevant optical parameters for each component. These parameters include, but are not limited to, the refractive index at different wavelengths, the type and degree of scattering, and the level of absorption. Additionally, the characteristics of the light sources and detectors can be specified as needed.

OpticStudio is an optimization software that allows users to find optimal solutions for optical systems with known and unknown characteristics. Users can define an optimization function and set constraints to guide the software in solving their problem. The optimization process in OpticStudio relies on ray-tracing, which can also be used independently as an analysis tool. The software offers two modes of operation: sequential and non-sequential.

In sequential mode, the ray-tracing simulation considers only the rays that travel from one object to the next, as defined by the editor. Rays cannot travel backward to previously defined objects or strike the same object more than once. This mode requires the optical system to be designed according to specific rules, enabling faster simulations by eliminating rays that do not adhere to these constraints.

In non-sequential mode, rays are free to move through space from any object to any other object, with the possibility of striking the same object multiple times. This mode provides a more realistic simulation but at the cost of increased computational effort. Since the primary objective was to simulate the human eye and evaluate its reflections, where rays must traverse different parts of the eye and potentially intersect the same surface multiple times, the human eye was modeled using the non-sequential mode.

**Figure 1:**
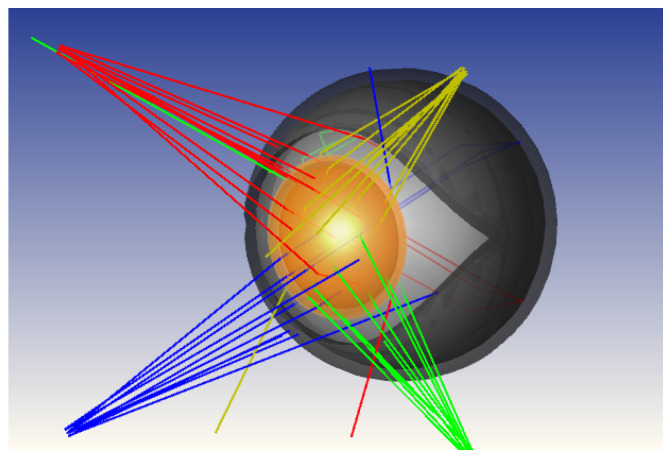
Non-sequential OpticStudio model of the human eye with four near-infrared light sources used for simulation-driven optimization of emitter and detector placement. The model includes the main anatomical components relevant to infrared light propagation and reflection. Lines with the same color correspond to rays coming from the same LED. Images used courtesy of ANSYS, Inc.

A non-sequential model of the human eye already exists in OpticStudio, but it lacks some components crucial to our goal and is defined only for visible light, while we are working in the IR part of the light spectrum. Regarding the missing parts, such as the eyelid, these were designed in 3D, and appropriate materials and properties were applied to them. Regarding the correct optical parameters for IR wavelengths, especially the refractive index, absorption, and scattering, they were defined and applied to all parts and materials involved to ensure an accurate response for the wavelength we are working with [22]. After all the parts were faithfully reproduced in terms of spatial measures, spatial placement, and response to infrared light, the human eye model consisted of the cornea, the anterior chamber, the vitreous chamber, the iris, the lens, the sclera, and the external lid. The full model is depicted in Figure 1.

In order to accurately model the refractive behavior of the various components of the eye with respect to IR light, we used the following equation from Conrady [23]:

(1) $$n\left(\lambda \right)=A+\frac{10^{2}\cdot B}{\lambda }+\frac{10^{9}\cdot C}{\lambda^{3.5}}$$

Using this formula, we modeled the IR dispersion of the aqueous humour, the cornea, the lens, and the vitreous humour using the parameters in Table 1.

**Figure 2:**
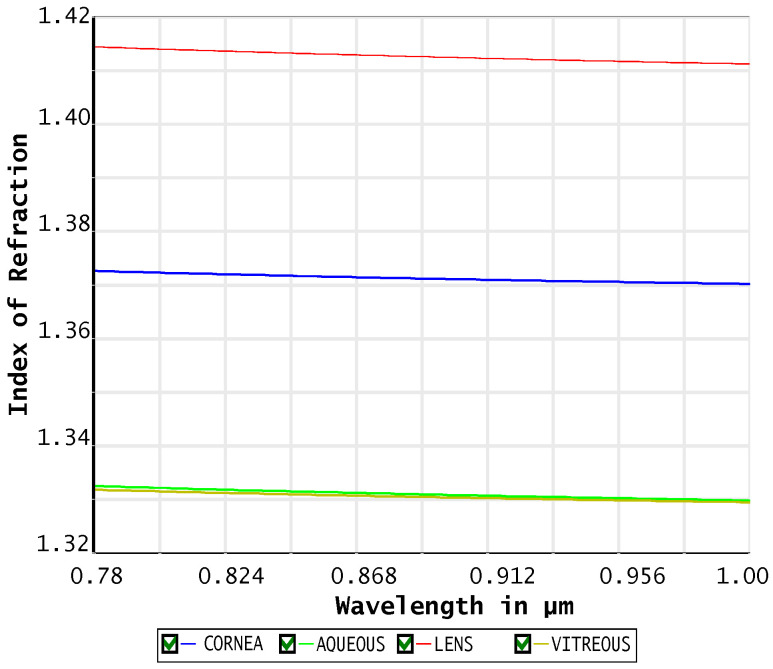
Refractive index dispersion of the main transparent eye components in the near-infrared (NIR) region. Images used courtesy of ANSYS, Inc.

As can be seen in Figure 2 the refraction index in the NIR region is almost constant for all the transparent components of the eye, so we adopted the values at 850 nm—that is, the central frequency of the adopted LEDs—for all of them.

As for the behavior of the non-transparent surfaces of the eye and surrounding areas (sclera, iris, eyelids, brow arch, etc.), they reflect incident light differently depending on the angle of incidence and the shape of the light beam. Specifically, the surface albedo [24] of the different surfaces was considered, which is the ratio between the radiosity (leaving flux emitted from a surface) and the irradiance (radiant flux received by a surface) [25]. This value can range from 0, the value of a black body that absorbs all incident radiation, to 1 for a perfectly reflecting body that reflects all incident light. Additionally, part of the incident light is reflected specularly (“glossy component”) and part diffusely (“matte component”) following the Lambertian behaviour [26]. The sclera, which is the primary non-transparent material that forms the outer surface of the eye and comes into contact with the eyelid while enclosing all other components of the eye, had a diffuse reflection coefficient of 0.55 and a specular reflection coefficient of 0.45. The modeling of these surfaces in the 3D rendering software was achieved using the Bidirectional Reflectance Distribution Function (BRDF) [27].

We paid particular attention to positioning the photodiode receivers to avoid capturing the directly reflected specular component, which could saturate the detector or, at the very least, reduce the signal variation between more reflective areas (e.g., sclera) and more absorbent ones (e.g., iris or pupil).

**Figure 3:**
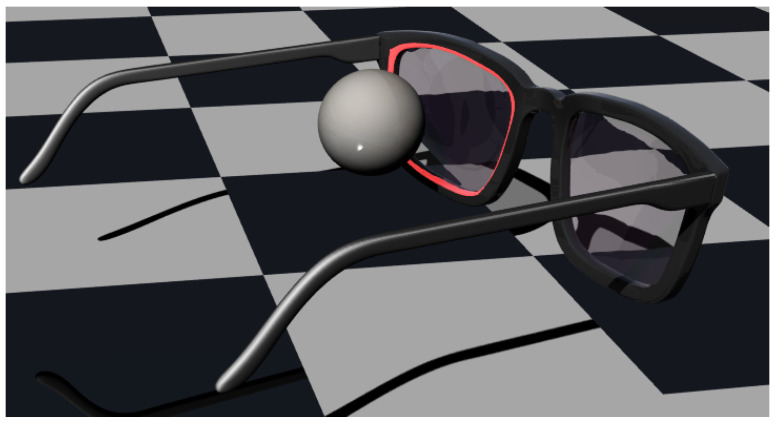
Rendering of the eye–eyewear configuration showing the spatial relationship between the eye and the half-frame. The red region highlights the available area for positioning near-infrared LEDs and photodiodes along the frame–lens interface.

The light detector was modeled as a virtual rectangle centered with respect to the pupil and positioned on a plane normal to the gaze direction when the eye is looking straight ahead. This plane was placed at a distance from the pupil equal to that of a common eyewear lens, approx. 13 mm. After the simulation, the data collected on the surface area of the virtual rectangular detector were intersected with the lens border of an eyewear half-frame. The optimal placement for the detectors on the prototype was then chosen along that stripe. Figure 3 shows a rendering of the eyeglass frame configuration relative to the eye, with the stripe highlighted in red indicating the available space for placing the IR detectors.

The IR sources were also placed in the same red region, and their orientations were set to point toward the corneal limbus (the border between the darker region of the cornea and the lighter region of the sclera) when the eye is looking straight ahead. This was done to maximize the light variation in response to eye rotations.

**Figure 4:**
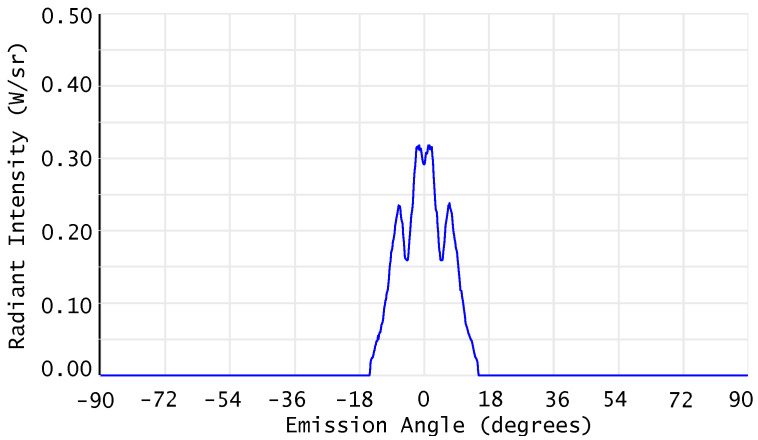
Angular radiation pattern of the near-infrared LED modeled in OpticStudio, configured to match the emission characteristics of the Lite-On HSDL-4260 device used in the prototype. Images used courtesy of ANSYS, Inc.

The light sources were configured to behave like the LEDs used in the real prototype (Lite-On HSDL-4260, by Lite-On Technology Corporation, Taipei, Taiwan) and emit light with the radiation characteristics shown in Figure 4.

Given a specific configuration regarding the position and orientation of the IR sources for the simulations, as described above, assuming *N* is the number of sensors, *L* is the number of IR light sources, *P* is the (discretized) positions of the sensors along the frame border and *M* is the number of all evaluated yaw and pitch rotations of the eye, we define $x_{n,l}\left(p,m\right)$ as the intensity acquired by the n-th sensor in the p-th position from the l-th source when the eyeball is in the m-th position. We then define

$${\bar{x}}_{n,l}\left(p,m\right)=x_{n,l}\left(p,m\right)-\frac{1}{M}\sum_{m=1}^{M}x_{n,l}\left(p,m\right)$$

the normalized intensity of the n-th sensor and then group the signals from all sensors associated with a specific source into a single vector in an *N*-th dimensional space:

$$x_{l}\left(p,m\right)={\left[\begin{array}{ccc} {\bar{x}}_{1,l}\left(p_{1},m\right) & \ldots & {\bar{x}}_{N,l}\left(p_{N},m\right) \end{array}\right]}^{⊺},$$

where $p={\left[\begin{array}{ccc} p_{1} & \ldots & p_{N} \end{array}\right]}^{⊺}$ represents the positions of the *N* sensors.

Since the contribution of each light source is independent of the others as described below, given an angular configuration *m* of the eyeball, we can define the vector $x\left(l,p,m\right)$ containing the contributions of all the *L* IR sources and all *N* sensors as follows:

$$x\left(l,p,m\right)={\left[\begin{array}{ccc} x_{1}{\left(p,m\right)}^{⊺} & \ldots & x_{L}{\left(p,m\right)}^{⊺} \end{array}\right]}^{⊺}$$

The total Euclidean distance between all the vectors $x_{l}$ belonging to the constellation of *M* eyeball configurations can be defined as follows:

(2) $$D\left(l,p\right)=\frac{1}{M\left(M-1\right)}\sum_{i=1}^{M}\sum_{\begin{array}{c} j=1\\ j\neq i \end{array}}^{M}∥x\left(l,p,i\right)-x\left(l,p,j\right)∥$$

The value of the maximum total distance *D* among all the possible combinations of *N* sensors in *P* positions results in the optimal configuration of the receivers for the chosen configuration of the *L* sources.

**Figure 5:**
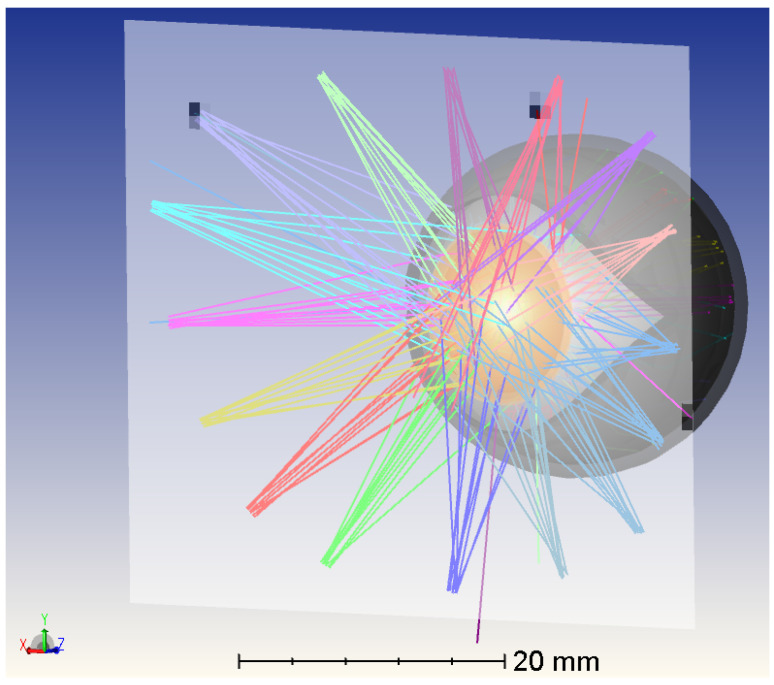
OpticStudio simulation of the eye model with a configuration of 16 near-infrared light sources placed on the detector plane. Lines with the same color correspond to rays coming from the same LED. Images used courtesy of ANSYS, Inc.

Numerous simulations were carried out with different quantities *L* of sources placed in various positions and different quantities of sensors *N* evaluated at different locations along the edge between the frame and the lens. For example, in Figure 5, a simulation with L=16 sources can be seen.

The simulations in OpticStudio were automated with MATLAB R2023b (MathWorks, Inc., Natick, MA, USA). [28] in order to accelerate the process to simulate all possible LEDs configurations according to different eyeball orientations.

Concerning the positioning of photodetectors, for each angular configuration *m* of the eye, a single LED was placed in a given position $\bar{l}$ along the perimeter of the frame, and a simulation was conducted by acquiring the light intensity received at each point at which the photodetectors can be placed $x_{\bar{l}}\left(p,m\right)$.

Since, for the usable light intensities and acquisition times, there are no significant non-linearity or phosphorescence effects from the surfaces of each part of the eye, the principle of the superposition of effects holds. Therefore, the signal acquired by a photodiode due to the contributions of multiple LEDs can be calculated simply as the sum of the contributions from each individual LED.

Analyzing the results of the different simulations, we found a compromise that provided good distinguishability between the different angular configurations of the eye, with an high value of *D* (Equation (2)): a system with four IR LED sources and four IR detectors, which was then implemented in the real model.

After all the LED subsets were processed and their related photodetector position was found, the best combination of the four LEDs was selected by looking at the mutual variation metrics of the related photodiodes’ optimization and selecting the LED combination that returned the highest result. In this way we were able to find the best LED placement and the related photodetector position.

The optimization of the sensor–source constellation using the distance metric *D* (Equation (2)) was performed based on a representative anatomical model of the human eye and nominal geometrical parameters of the eyewear system. It is acknowledged that inter-subject anatomical variability, such as differences in eye geometry, surface reflectance, or pupil size, as well as mechanical tolerances in lens positioning and sensor placement, may affect the absolute intensity values measured by the photodetectors. However, the proposed optimization criterion is based on the relative separation between signal vectors associated with different gaze directions rather than on their absolute magnitude. As a result, global variations in reflectance or moderate geometric deviations are expected to primarily introduce scaling or offset effects in the acquired signals, while preserving the relative structure of the constellation used for gaze discrimination. Furthermore, the final gaze estimation was performed using data-driven methods that rely on subject-specific calibration, which compensates for individual anatomical differences and systematic hardware variations. For these reasons, the optimized configuration obtained from the simulation framework provides a practical and generalizable solution for sensor and source placement, while subject-specific and system-specific variations are addressed during the calibration and estimation stages.

### 2.2. Evaluation System

With optimal positioning and tilting of the LEDs and photodetectors, a prototype eye-tracking eyewear was built. Since this prototype has not been certified as eye-safe, it could not be tested on humans. Therefore, synthetic models of the human eye and a machine to move them in front of the prototype were needed. This setup not only allows for the safe testing of the device but also enables the creation of a large dataset of measurements with a known ground truth of the eye’s location.

Two different synthetic models of the human eye were used. The first was a rougher model, consisting of a plastic doll’s eye, while the second was a more refined model called OEMI-7. The OEMI-7 eye model is a realistic eye model designed for ocular fundus imaging which has already been used to validate eye-tracking systems, e.g., in [29,30]. This eye model, according to the manufacturer’s datasheet, incorporates a simplified anterior chamber, crystalline lens, and fundus, along with features such as an optic disc, blood vessels, simulated retinal detachment, and fluorescent structures for imaging experiments. These characteristics make the model suitable for controlled optical evaluation and sensor–source geometry optimization, as it provides a stable and repeatable representation of the macroscopic geometry and first-order optical behavior of the human eye. However, as specified in the OEMI-7 documentation, the model does not reproduce several physiological characteristics of real human eyes, including micro-scale surface texture, tear-film dynamics, moisture-related reflections, pupil size variability, and eyelid motion. Consequently, a transfer gap between synthetic and real eyes is expected; in the proposed system, this gap is mitigated by relying on relative spatial variations in reflected infrared light and by employing subject-specific calibration during the estimation phase.

The machine used to move the eye is based on the Quanser 3-DOF gyroscope, a device that we modified and fitted for our purposes. Specifically, we adapted this device to serve as a high-precision gimbal set. Thanks to the power and accuracy of the rotations provided by this device, it is possible to realistically simulate the saccadic and micro-saccadic movements of the eyeball, as well as the sudden transitions between fixation points.

The 3-DOF gimbal consists of a disk mounted inside an inner gimbal, which is in turn mounted inside an outer gimbal. The entire structure is supported by a rectangular frame that can rotate about its vertical axis using a slip ring design. The gimbals are also equipped with slip rings, allowing them to rotate freely and providing the central disk with three degrees of freedom. The system is equipped with four DC motors and four 12-bit encoders, enabling the precise control of individual axes. Axis positions are measured using high-resolution optical encoders. Although the gimbals and outer frame are free to rotate, the system allows for the fixation of any desired axis (outer frame, red and blue gimbals).

The device was modified for our purposes to allow the eye to move only around the yaw and pitch axes. For this reason, the blue inner gimbal and the golden disk mounted within it were removed. The red outer gimbal was partially disassembled to accommodate and fix a custom 3D-printed adapter where the eye model was mounted. With this modification, the center of the eye aligns perfectly with the yaw and pitch movement axes provided by the silver frame and the red gimbal, respectively.

**Figure 6:**
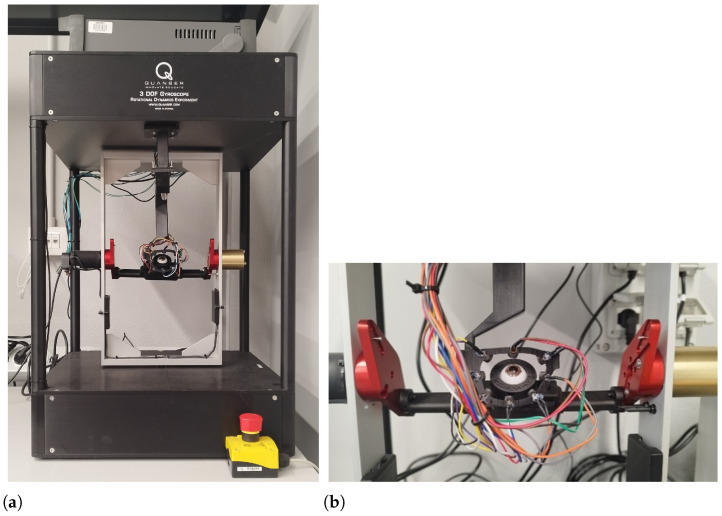
Experimental evaluation setup. (**a**) Modified Quanser 3-DoF gyroscope used as a high-precision gimbal for controlled eye rotations in yaw and pitch. (**b**) Detail of the OEMI-7 synthetic eye and the half-frame eye-tracker prototype mounted on the gimbal.

The eyewear half-frame was attached to the gimbal through a 3D-printed adapter and a platform that allows movements along the x, y, and z axes to allow for fine adjustments, ensuring the half-frame was positioned correctly in front of the eye. The modified gimbal and a detail of the OEMI-7 eye model mounted on it, along with the half-frame eye-tracker prototype, are depicted in Figure 6a and Figure 6b, respectively.

The gimbal-based setup provides precise and repeatable control of eye rotations in yaw and pitch, enabling isolated evaluation of the proposed PSOG sensing architecture and estimation algorithms. However, it does not model several factors present in real smart eyewear usage, including frame translation and slippage, facial-geometry constraints, relative motion between the eye and the frame, and head–eye coordination. These effects represent important sources of error in wearable eye-tracking but are intentionally excluded here to allow for controlled validation of the optical design and algorithmic behavior. Their impact will be addressed in future work through experiments on human participants and the development of calibration and compensation strategies.

**Figure 7:**
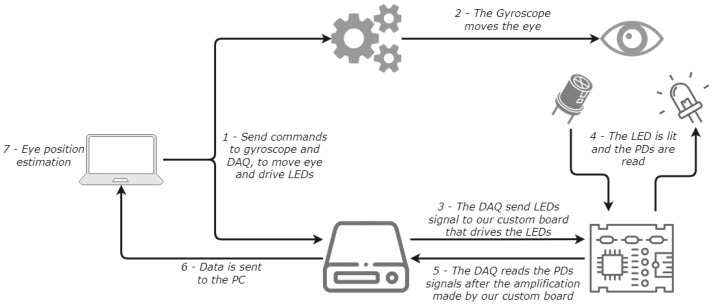
Block diagram of the experimental setup and data acquisition system, including LED modulation, photodiode signal conditioning, gimbal control, and synchronized data acquisition.

The part regarding the driving of the LEDs and the reading of photodetectors is managed by a NI DAQ USB-6353 (National Instruments, Austin, TX, USA), which sends the modulating waveform to the LEDs and reads the photodetector signals. An additional PCB is used between the DAQ and the LEDs/photodetectors to amplify the signals. The gimbal movements are controlled using MATLAB with a PID controller; LEDs and the photodetector signals are also managed using a MATLAB script. Acquired and generated signals, together with gimbal movements, can be controlled simultaneously using a MATLAB script used for dataset acquisition and algorithm testing. A block diagram of the experimental setup described is presented in Figure 7. While this laboratory setup is not representative of a wearable implementation in terms of power consumption, the first high-level system power estimates for an embedded eyewear platform are reported in Section 3.

### 2.3. Datasets and Signals

In order to simultaneously detect the amount of light received by each of the *N* detectors and emitted by each of the *L* sources, modulators $h_{l}\left(t\right)$ that were all orthogonal to each other during the acquisition period *T* were used. This means that, given two modulators $h_{i}\left(t\right)$ and $h_{j}\left(t\right)$, the following relationship holds:

$$\int_{T}h_{i}\left(t\right)h_{j}\left(t\right)dt=\left\{\begin{array}{cc} 0 & i\neq j\\ 1 & i=j \end{array}\right.$$

This approach allows, for each receiver, for the isolation of the contribution of each source using a demodulation approach analogous to the matched filter [31]. In particular, if $a_{l}$ is the constant amplitude of the l-th source and $b_{l}$ is the amplitude of the modulating function (with $b_{l}<a_{l}$), the emitted radiation will be $a_{l}+b_{l}h_{l}\left(t\right)$ and the reflected part from the eye and captured by the n-th photodiode will be ${\hat{a}}_{l,n}+{\hat{b}}_{l,n}h_{l}\left(t\right)$. Since the sources and receivers are synchronous, no delay is present and the contribution of the l-th source can be obtained as

$$\left({\hat{a}}_{l,n}+{\hat{b}}_{l,n}h_{l}\left(t\right)\right)*h_{l}\left(-t\right)=\int_{T}{\hat{b}}_{l,n}h_{l}\left(t\right)\cdot h_{l}\left(t\right)dt={\hat{b}}_{l,n}$$

In the case of multiple parallel sources the signal received from the n-th photodiode will be

$$x_{n}\left(t\right)=\sum_{l=1}^{L}\left({\hat{a}}_{l,n}+{\hat{b}}_{l,n}h_{l}\left(t\right)\right)=\sum_{l=1}^{L}{\hat{a}}_{l,n}+\sum_{l=1}^{L}{\hat{b}}_{l,n}h_{l}\left(t\right)$$

but, thanks to the orthogonality condition of the modulating functions, we can extract each single contribution ${\hat{b}}_{l,n}$ through demodulating with the specific function $h_{l}\left(t\right)$:

(3) $${\hat{b}}_{l,n}=x_{n}\left(t\right)*h_{l}\left(-t\right).$$

Various modulating functions that are orthogonal to each other during the integration period *T* can be employed. In particular, for sinusoidal functions, we can use

$$h_{l}\left(t\right)=\sqrt{\frac{2}{T}}\cos\left(2\pi \frac{k_{l}}{T}t\right)$$

with $k_{l}\in N$ while, for square waves with a duty cycle of 50%, we can use

$$h_{l}\left(t\right)=\frac{1}{\sqrt{T}}\mathrm{sgn}\left(\cos\left(2\pi \frac{k_{l}}{T}t\right)\right);$$

where sgn() is the “sign” function.

To implement the system, a discrete-time realization was chosen to allow for greater efficiency, compactness, and noise robustness with respect to the continuous (analog) one. To this end, we defined a sampling period of 12.8 kHz for the photodiodes, and the LEDs were modulated with four carriers at 400, 800, 1600, and 3200 Hz.

Ambient infrared illumination is typically dominated by broadband or low-frequency components (e.g., sunlight and artificial lighting flicker), and the presence of narrowband ambient interference that is precisely aligned with the selected carrier frequencies is therefore unlikely. Nevertheless, discrete-time sampling, finite integration windows, and the use of square-wave modulation introduce non-ideal orthogonality and harmonic components, which can result in residual spectral leakage and inter-channel interference. In addition, dynamic ambient infrared conditions may further affect demodulation performance. These effects are not fully mitigated in the current prototype and represent an important consideration for future robustness improvements, such as enhanced filtering, adaptive background estimation, or alternative modulation strategies.

**Figure 8:**
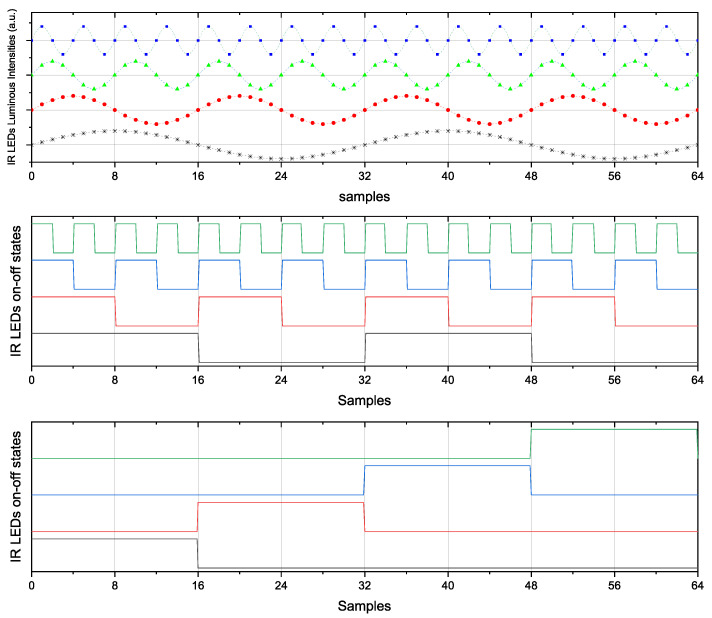
The signals emitted by the LEDs in the three considered scenarios. A different color line is depicted for each different LED. $V_{max}$ and $V_{min}$ are the maximum and minimum voltages applied to the LEDs to make them follow the desired signal. For the constant signal, all the samples are shown, while for the sinusoidal and the square wave signal, given their periodicity, only the first 64 samples are shown for a better visualization.

Given an acquisition time $T_{acq}=5$ ms, we can obtain 2 periods of the first sine or square wave, 4 for the second, 8 for the third and 16 for the fourth one; while, for the sequential approach, we just collect 16 samples from each source, resulting in a total of 64 samples for each wave, as depicted in Figure 8. Using the gimbal and the human eye model, we can acquire datasets with different configurations and varying external noise. These datasets contain raw data from the photodetectors when the eye, during fixations, looks in different directions. This allows us to collect data on the various fixation points of the eye, which is essential for developing and testing eye-tracking algorithms.

During the acquisition of the datasets, the eye was moved between −20° and +20° in yaw with a step size of 1°, and between −20° and +20° in pitch with a step size of 1°. At each new fixation point, the LEDs emit the predefined signals, and the photodetectors begin to receive IR light, allowing the information to be sampled. The gimbal then moves to the next point.

In total, we acquired data on 1681 different fixation points within the considered area. Saccades or microsaccades are not part of the datasets.

**Figure 9:**
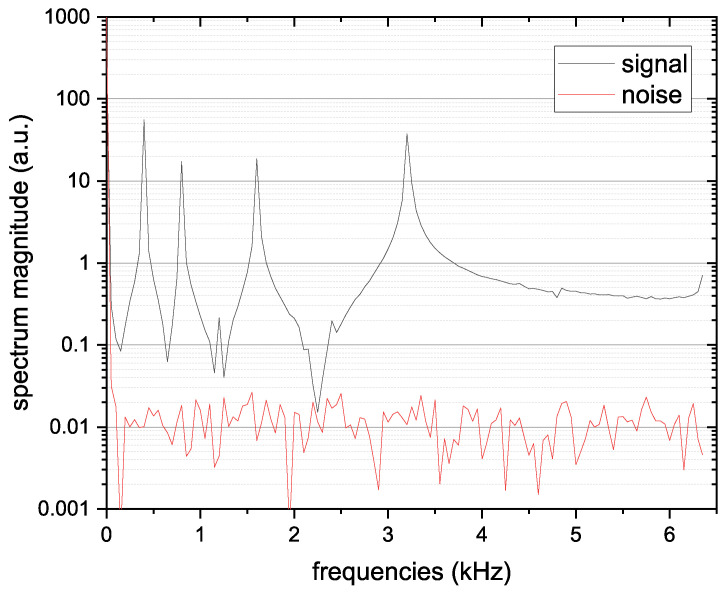
Frequency-domain representation of the photodiode signal with LEDs on (signal) and off (ambient noise), showing clear spectral peaks at the modulation frequencies (400, 800, 1600, and 3200 Hz).

We adopted three different types of signals: sinusoidal waves, square waves at the aforementioned frequencies, and sequential on–off switching of each LED. The latest solution avoids potential cross-talk between the different signals delivered in parallel but, at the same time, it is also slower at the same signal-to-noise ratio because the individual readings occur sequentially rather than in parallel, as in the other two cases. This can result in greater inaccuracy in tracking saccades and microsaccades. It is important to note that due to non-linearities in the signal generation and acquisition process, there can be spectral leakage that causes partial overlap between the acquired signals. This is visible in Figure 9, where for each modulating frequency, there is a widening of the expected impulse, reducing the accuracy of the amplitudes detected and associated with the contribution of each source. By adopting the sequential solution, that is, removing the temporal overlap between the different LED signals, this issue is avoided.

The signals emitted from the LEDs in each of the three aforementioned cases are depicted in Figure 8.

Thanks to the orthogonality required between the modulating signals of each LED and the synchronization between modulating and demodulating signals, by simply adopting Equation (3), it is possible to directly extract the contribution of each LED. In order to analyze the entire spectrum of the received signal, including the potential presence of noise localized at certain frequencies or frequency intervals, one can evaluate the Discrete Fourier Transform (DFT) for sinusoidal waves or the Haar transform for square waves: In this way, it is possible to identify frequencies with high ambient noise (consider, for example, the harmonics of 50 Hz present in indoor lighting with incandescent or fluorescent lamps) and avoid such frequencies for the modulation of the sources. In order to evaluate the power of light noise, for each of the approaches described above, the signal from each photodetector is also periodically acquired with all sources turned off. This makes it possible to evaluate the overall noise power as well as, through the aforementioned transforms, evaluate its spectrum at different frequencies to locate any peaks to avoid as frequencies for the modulating sources. This will also allow for a reduction in the power emitted by the LEDs in low-IR ambient light conditions, thereby reducing the battery’s energy consumption.

**Figure 10:**
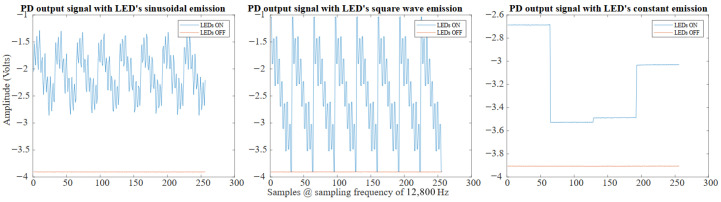
Time-domain signals acquired by a single photodiode for sinusoidal, square-wave, and constant LED emission schemes, shown for both LED-on and LED-off conditions.

So, each PD produces a vector of 4 features and since we have 4 PDs in total we have a vector of 16 features that could be used to estimate the eye position. After the acquisition of each position, both during the acquisition of the datasets but also in the next algorithm testing phases, a reading of the PDs with the LEDs off is performed to obtain data on the background noise. This data is transformed into a vector of 16 features, as well as the data with LEDs on, and subtracted from the total. The raw signals received by a single PD are depicted in Figure 10. The result of the FFT applied to the signal received by a single PD when the LEDs are on/off and the eye looks in a certain direction is depicted in Figure 9.

In the case of square wave signals, the situation is similar to the sinusoidal case. The square wave are emitted together, and at the photodetectors their sum is received. An acquisition with LEDs off is subtracted to an acquisition with LEDs on to remove the background noise. The raw signals received by a single PD are depicted in Figure 10. To demodulate the signal received by the photodetectors, a matched filter is used. Using the matched filter principle, it is possible to isolate the contribution of each LED in each photodetector. For example, the signal on the photodetector *x* due to the LED *y* in a period T of this LED, which can be obtained as follows:

(4) $$S_{LED_{y}}\left[t_{0},t_{0}+T\right]=\int_{\begin{array}{c} LED_{y}^{ON} \end{array}}S_{PD_{x}}\left(t\right)dt-\int_{\begin{array}{c} LED_{y}^{OFF} \end{array}}S_{PD_{x}}\left(t\right)dt$$

And a digital version on the above equation is

(5) $$S_{LED_{y}}\left[n_{0},n_{0}+T\right]=\sum_{\begin{array}{c} LED_{y}^{ON} \end{array}}S_{PD_{x}}\left(n\right)-\sum_{\begin{array}{c} LED_{y}^{OFF} \end{array}}S_{PD_{x}}\left(n\right)$$

The contribution of the LEDs at higher frequencies (multiples based on powers of 2 of the current LED frequency) is added equally both to the $LED_{y}^{ON}$ and $LED_{y}^{OFF}$ parts and therefore, their contributions cancel each other out when the difference is taken. Similarly, the contribution of the LEDs at lower frequencies (submultiples based on powers of 2 of the current LED frequency) is also added equally to both the $LED_{y}^{ON}$ and $LED_{y}^{OFF}$ parts, and therefore, this contribution is canceled out by taking the difference. It is crucial that the integrals are performed in phase with the LED signal, but synchronizing the LEDs and photodiodes should not pose a problem. Once the contribution of each LED to each photodetector is understood, four features representing the intensity of the four LEDs’ contributions can be extracted from the signal of each of the four photodetectors. This process forms a 16-feature vector corresponding to the current eye position.

In the case of constant signals, each LED stays on for 5 ms while the others are off. The LEDs turn on, alternating one after another, and thus covering the 20 ms. Once the signal is acquired, the acquisition with LEDs off is subtracted from the acquisition with LEDs on to remove the background noise. The raw signals received, by a single PD are depicted in Figure 10. In this case, for each PD the 4 features are calculated as the mean intensity received by each LED when the interested LED was on, giving us a vector of 16 features.

In all the cases and for each signal type used, the vectors of 16 features will be the input of our proposed eye-tracking algorithms.

### 2.4. Algorithms

Different approaches involving linear and non-linear regression, supervised learning, artificial intelligence, or neural networks can be used to estimate the yaw and pitch positions of the eye.

The first approach evaluated is a simple and lightweight algorithm based on the concept of a Look-Up Table. Starting with a training dataset containing data acquired when the eye looks at all positions of a predefined grid (ranging from −20° to +20° on both the yaw and pitch axes, with a 1° step), a table is created to store the 16-element vectors extracted from the data at each training position. This table has the same dimensions as the grid and stores a 16-element vector corresponding to each grid position at the corresponding coordinates in the table. To improve reliability and robustness, multiple datasets acquired on the same grid can be used, with the table storing the mean of the 16-element vectors corresponding to each position. Once the table is filled, the estimation can occur in three different ways:

- Coarse: This mode selects the entry in the table with the closest vector values and outputs it.
- Full: This mode selects *n* entries in the table with the data most similar to the position estimates. It then performs linear interpolation between their positions, using the error between their data as weights, to obtain a single position estimate.
- Neighbors: This mode first selects the entry in the table with the data most similar to the position estimate. It then considers the *n* nearest points in the table to the chosen entry. Finally, it interpolates between the positions of these selected points using parabolic interpolation in the x and y directions to produce the final estimate.

**Figure 11:**
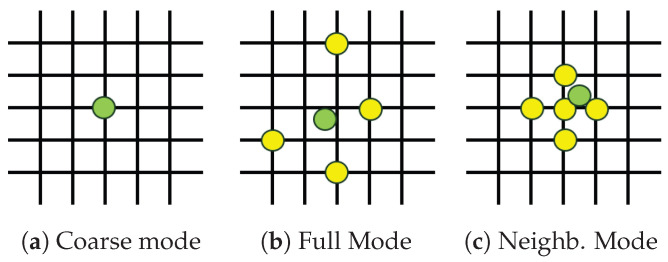
Visualization of the three Look-Up Table estimation modes: (**a**) coarse, (**b**) full interpolation, and (**c**) neighbor-based interpolation. The estimated gaze position is shown in green, while the reference positions used for estimation are shown in yellow.

Figure 11 shows a visualization of the points used for estimation in each case.

The second approach involves using a shallow neural network (NN). The main advantages of such a network are its constrained training and inference time, as well as its limited resource usage. The network takes the feature vector as input and processes it with a fully connected layer with an output size of 50. The result is then passed through a ReLU activation function, and finally, the output is processed by another fully connected layer with an output size of 2, representing the yaw and pitch estimations. This approach is applied in two different ways:

- NN: The network described above takes a 16-feature vector as input and produces an estimate.
- NN + PCA: The vector of 16 features is preprocessed using Principal Component Analysis (PCA) to achieve lower dimensionality and noise reduction while preserving most of the data’s variability (information). The PCA is derived from the same training set used for the network, and the number of Principal Components (PCs) retained is chosen to ensure that 95% of the original variance is maintained. Consequently, the initial 16-feature vector is transformed into a vector with n<16 features, which is used as the input to the network defined above for estimation. This approach can facilitate network training, improve training and inference times, and enhance performance.

The two networks, i.e., one network without and one with PCA, were trained on the same training dataset used for the Look-Up Table algorithm.

The final estimation method tested is based on Gaussian Process Regression (GPR) [32]. GPR is a powerful non-parametric Bayesian approach to regression that offers a probabilistic framework for modeling complex relationships between variables. Unlike traditional regression methods, which assume a specific functional form for the relationship between inputs and outputs, GPR defines a distribution over functions directly. This allows it to capture intricate patterns and uncertainties inherent in the data. The core of GPR is the Gaussian process, characterized by its mean function and covariance function (or kernel). The choice of kernel function is crucial, as it encodes assumptions about the smoothness, periodicity, and other properties of the function being modeled. Given a set of training data, GPR uses the covariance function to compute the joint distribution of the observed data and the values at new input points, enabling predictions that include both a mean estimate and a measure of uncertainty. This approach not only provides predictions but also quantifies the confidence in those predictions. In this work, two different Gaussian Process Regression (GPR) models are trained on the same training dataset used for the previous approaches, employing the Matern 52 kernel. The first model is designed for yaw estimation, while the second model is for pitch estimation. Both models use the same 16-feature vector as the input and generate the two required estimations.

Finally, other estimation methods, such as a linear regression with and without the PCA and the GPR with PCA were tested, but these are not included in this work due to their lack of performance. The choice of these algorithms was driven not only by estimation accuracy but also by practical deployment considerations. The target application involves embedded and potentially wearable systems, where constraints in terms of available memory, computational resources, power consumption, and real-time execution are critical. More complex machine learning models, such as deep neural networks or large-scale kernel-based methods, typically require significantly higher memory footprints and longer inference times, and often rely on hardware acceleration or high-precision floating-point operations, which are not always available on resource-constrained platforms. In contrast, the selected approaches, namely the Look-Up Table method, shallow neural networks, and Gaussian Process Regression, offer a favorable trade-off between performance and computational efficiency. In particular, shallow models enable predictable and low-latency inference, are easy to implement in microcontrollers or low-power processors, and offer improved robustness in real-time operation. Given these considerations, the investigated methods are well-suited for practical, deployable eye-tracking systems, while more complex models would introduce unnecessary implementation complexity without guaranteeing proportional performance gains.

## 3. Results

To test the proposed estimation methods, a test dataset was acquired for all three types of emission signals. The test dataset consists of 50 randomly sampled gaze positions within the same angular range as the training grid. Due to the dense sampling of the training grid, strict independence between training and testing samples is not enforced in this evaluation. As a result, the reported errors, particularly for non-parametric methods such as Gaussian Process Regression and lookup-table approaches, may reflect the partial interpolation of the underlying synthetic relationship rather than independent generalization performance. To quantify the degree of interpolation induced by the dense 1° × 1° training grid, we computed, for each test point, the Euclidean distance to the nearest training-grid sample. That distance ranged from 0.042° to 0.673°, with a mean 0.353° and median 0.364°, confirming that the test set lies close to the training grid and that the GPR and LUT results may partially reflect interpolation.

This test set is intended to provide a preliminary assessment of estimation behavior under controlled conditions rather than a statistically exhaustive evaluation. While this sampling density is sufficient to compare the relative performance of different estimation methods, it does not allow for the high-confidence statistical characterization of error distributions.

The raw data from the test dataset was processed in the same manner as the training dataset to extract the 16-feature vector. This vector then enables inference using the three different modalities: the Look-Up Table, the Neural Network (both with and without PCA), and the Gaussian Process Regressor.

**Figure 12:**
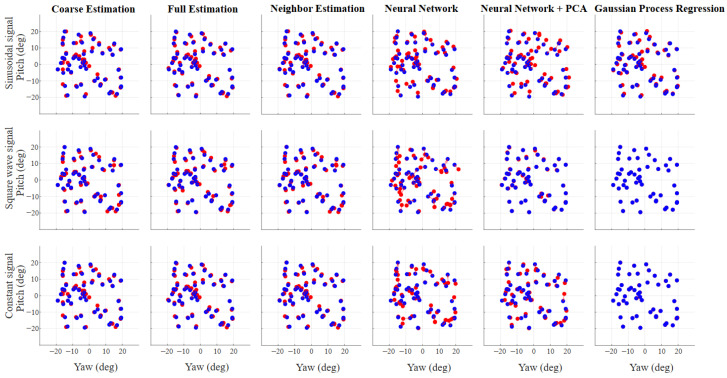
Qualitative results of gaze estimation on the test dataset for all emission signals and estimation methods. Blue points represent ground-truth gaze positions, while red points indicate the corresponding estimates.

Figure 12 shows the qualitative results of the inference on the test dataset of 50 random positions obtained using the six estimation approaches. Table 2 reports the Root Mean Squared Error (RMSE) and the Mean Inference Time (MIT) for these six estimation approaches on the same test dataset. The RMSE is defined as

(6) $$\mathrm{RMSE}\left(x,y,\hat{x},\hat{y}\right)=\sqrt{\frac{\sum_{i=1}^{N}{\left(x_{i}-{\hat{x}}_{i}\right)}^{2}+{\left(y_{i}-{\hat{y}}_{i}\right)}^{2}}{N}}$$

where *N* is the number of points in the test dataset, *x* and *y* are the vectors containing the yaw and pitch ground-truth positions, $\hat{x}$ and $\hat{y}$ are the vectors containing the yaw- and pitch-estimated positions. While RMSE provides a compact summary of average estimation accuracy, it does not capture worst-case behavior or error tails, which are critical for interface reliability. These metrics are not reported in the present study and will be considered in future evaluations involving larger test sets and repeated trials. The MIT was instead defined as

(7) $$\mathrm{MIT}\left(A,F\right)=\frac{\sum_{i=1}^{N}T\left(A\left(f_{i}\right)\right)}{N}$$

where *N* is the number of points in the test dataset, *F* is the matrix containing the 16 feature vectors of the test dataset points on its rows $f_{i}$, *A* is the algorithm selected to estimate the position of the current feature vector, and *T* is a function to measure the time needed to *A* to produce the estimate. It should be noted that this measure is influenced by the status of the machine where the code is executed. However, since the result is the average of 50 samples of execution times, and given that these samples showed minimal variability and that the six estimation methods were executed sequentially (ensuring the machine status was consistent), this measure can be considered reliable for assessing the order of magnitude of the time required for inference.

Regarding the three versions of the Look-Up Table, all three approaches perform similarly due to the dense sampling of the relevant range. However, the Coarse mode has limitations compared to the others, as it cannot generate predictions outside the sampling grid. The lookup-table approaches are evaluated under a fixed geometry and controlled acquisition conditions. As such, they do not account for calibration transfer, sensor displacement, or robustness to sensor shift, which are known limitations of grid-based methods. While lookup tables provide strong baselines under dense sampling and stable configurations, their performance is expected to degrade under changes in sensor placement or user geometry. These aspects are not addressed in the present study and represent important directions for future robustness analysis. In terms of computation time, despite the differences in the three modalities, where the Full and Neighbor modes involve more calculation steps than the Coarse mode, the Mean Inference Time (MIT) is similar across all LED emission types. This indicates that the Full and Neighbor modalities, while offering improvements over the Coarse version, can be implemented efficiently with minimal additional cost.

For the Neural Network without PCA, the RMSE results are acceptable but not as favorable as those from the Look-Up Table. This performance discrepancy may be attributed to the network’s architecture, which lacked complexity and thus struggled to generalize well on the test data. This explanation is supported by the fact that the Neural Network approach with PCA, where Principal Component Analysis is used as a preprocessing step, performs significantly better for both the Square Wave and Constant cases. PCA reduces the number of input features, which in turn simplifies the problem. Specifically, after PCA, the number of retained features *n* is 6 for all sinusoidal, square, and constant signal cases. However, this is not true for the Sinusoidal signal. The reduced performance observed for sinusoidal signals should not be interpreted as a general limitation of regression-based methods, but rather as a consequence of the specific feature representation and model architectures adopted in this work. More expressive architectures or alternative feature designs could potentially improve performance for sinusoidal trajectories, at the cost of increased computational complexity.

Regarding the computation time of the NN approach, it is important to note that the MIT measured here considers only the inference time and does not include the Neural Network training time. This method proved to be significantly slower than the others. However, the PCA preprocessing in the NN + PCA approach also contributes to a faster inference process.

The final approach, Gaussian Process Regression (GPR), delivers the best results in terms of RMSE for all three types of emission signals. The very low RMSE values obtained by GPR for square-wave signals should be interpreted with caution. Given the structured nature of the synthetic dataset and the partial proximity between training and testing samples, these results may reflect the near-interpolation of a regular relationship rather than robust generalization. While GPR provides a probabilistic framework, uncertainty estimates and repeated independent trials were not included in the present evaluation and will be necessary for a more rigorous assessment. In terms of computing times, GPR also ranks among the lowest of all approaches.

Overall, GPR with the Square Wave signal shows the best performance in terms of RMSE. Its RMSE is at least one order of magnitude better than that of other approaches and nearly an order of magnitude better than its own performance with the Constant signal, which ranks second. This performance is evident in Figure 12, where the estimated positions closely match the ground-truth positions. However, while GPR does not achieve the best Mean Inference Time (MIT), it comes in second, very close to the best MIT, which was obtained by the NN + PCA with the Constant signal.

In addition to RMSE, Table 3 reports the error distribution in terms of the 50th, 90th, and 95th percentile angular error and the maximum error, to better capture tail behavior on the 50-point test set. The percentile statistics confirm the trends observed from RMSE, while also highlighting tail behavior. For sinusoidal modulation the lookup-table and GPR methods show similar median errors (P50 ≈ 0.9–1.0°) with worst-case errors around 2–3°, whereas the NN-based models exhibit heavier tails (Max up to ∼6–8°). For square-wave and constant modulation, GPR (and NN+PCA for square waves) yields very small errors across percentiles; however, given the dense 1° training grid and the measured proximity of test points to the grid, these results should be interpreted as near-interpolation under controlled conditions rather than as a guarantee of real-world generalization.

In addition to inference timing, approximate power consumption was estimated based on a system representative of an embedded eyewear implementation, using NIR LED drive currents, duty cycles, and photodiode front-end power comparable to those reported in Pezzoli et al. [9] and Pettenella et al. [10]. Under these assumptions, the estimated total system power is on the order of 45 mW, of which 24 mW for the MCU consumption (STM32U5G9 by STMicroelectronics, Geneva, Switzerland), 15 mW for the analog front-end and 6 mW for the LEDs. Finally, the reported inference times are intended solely for a relative comparison of algorithmic complexity. In an embedded implementation, absolute latency would scale with the target microcontroller, and the associated computational power consumption would be directly related to inference time, while the power estimates reported here currently refer only to signal acquisition, as inference is not performed on-board.

## 4. Conclusions and Future Works

This work presented the design and experimental evaluation of a frame-integrated infrared photosensor oculography (PSOG) eye-tracking system intended for smart eyewear applications. The proposed architecture employs four near-infrared LEDs and four photodiodes per eye, integrated into the eyewear frame, with emitter and sensor placement optimized through simulation-driven analysis of ocular light reflection using OpticStudio. This approach enabled the systematic selection of component positions and orientations to maximize the information content of the acquired signals under wearable constraints.

Based on the optimized optical configuration, a functional prototype was developed and evaluated using the OEMI-7 artificial eye model mounted on a Quanser 3-DOF gyroscope. This setup allowed for the repeatable and controlled acquisition of PSOG signals across a defined range of yaw and pitch angles within the human field of view. The resulting datasets were used to train and evaluate multiple lightweight regression methods for gaze estimation, selected with embedded deployment, power efficiency, and computational simplicity in mind. Among the evaluated approaches, Gaussian Process Regression combined with square-wave LED modulation achieved the best overall estimation accuracy while maintaining a competitive inference time, highlighting a favorable trade-off between performance and computational cost in this controlled setting.

The results demonstrate the feasibility and potential effectiveness of the proposed PSOG architecture for gaze estimation under controlled conditions and validate the underlying design choices related to optical layout, modulation strategy, and algorithm selection. However, the reported accuracy and performance results were obtained under controlled geometry and illumination and do not account for real-world wearable error sources, such as frame slippage, eye–frame misalignment, facial geometry variability, or head–eye coordination. Additionally, the study deliberately focuses on a constrained experimental scope to enable repeatable characterization while avoiding confounding sources of variability.

Future work will focus on extending this feasibility study to a system which can be used in real-world scenarios. This includes validation on human participants to assess robustness to anatomical variability, eyelid dynamics, skin reflectance, and device slippage, as well as the development of calibration strategies that are suitable for everyday use. Additional efforts will address dynamic eye movements beyond fixation, including saccades, by refining both the signal acquisition pipeline and the feature representation. From a system perspective, further optimization of power consumption, sampling-rate trade-offs, and on-device implementation will be explored, including the investigation of analog or mixed-signal processing to increase temporal resolution and reduce digital processing overhead. Finally, future evaluations will emphasize strict separation between training and testing samples and more rigorous generalization analysis under independent conditions, particularly in the context of human-subject validation.

## Figures and Tables

**Table 1:** Coefficients of Conrady’s formula for different transparent eye parts.

Material	A	B	C
*Cornea*	1.36	6.67 × 10^−3^	3.87 × 10^−4^
*Aqueous Hum.*	1.32	8.47 × 10^−3^	2.32 × 10^−4^
*Lens*	1.40	9.39 × 10^−3^	3.93 × 10^−4^
*Vitreous Hum.*	1.32	6.73 × 10^−3^	3.34 × 10^−4^

**Table 2:** RMSE and MIT for all the signals and methods proposed. Those results are obtained on a test set of 50 random positions, executed on an Intel Core i9-13950HX processor.

		Coarse	Full	Neighbor	NN	NN + PCA	GPR
Sinusoid	RMSE (deg)	1.29	1.18	1.20	2.04	2.18	1.10
	MIT (ms)	1.79	1.75	1.69	117	0.630	0.811
Square	RMSE (deg)	1.31	1.17	1.31	3.38	0.429	**0.0249**
	MIT (ms)	1.81	1.86	1.72	22.5	0.294	0.200
Constant	RMSE (deg)	1.21	1.11	1.17	2.36	1.48	0.0928
	MIT (ms)	1.57	1.63	1.61	51.94	**0.174**	0.305

**Table 3:** Error distribution summary (degrees) on the 50-point test set: 50th, 90th, and 95th percentile angular error (P50/P90/P95) and maximum error (Max).

Signal	Method	P50	P90	P95	Max
Sinusoid	LUT (Coarse)	0.96721	2.0361	2.3237	2.8558
	LUT (Full)	0.89655	1.6841	2.0341	2.6728
	LUT (Neighbor)	0.96181	1.7546	1.8787	2.9516
	NN	1.4399	2.9146	3.6886	5.9814
	NN + PCA	1.9963	3.4311	3.7773	4.9244
	GPR	0.89903	1.7141	2.1512	2.1963
Square	LUT (Coarse)	0.97462	2.2210	2.3842	3.3026
	LUT (Full)	0.92230	1.8332	2.0469	2.3922
	LUT (Neighbor)	0.91122	2.0542	2.4982	3.1242
	NN	3.0598	6.0609	6.8529	7.9391
	NN + PCA	0.18172	0.62804	0.84177	0.95130
	GPR	0.017988	0.040706	0.041949	0.086146
Constant	LUT (Coarse)	0.96144	1.9259	2.3237	2.8558
	LUT (Full)	0.89643	1.6446	2.0508	2.6750
	LUT (Neighbor)	0.90849	1.7153	2.0115	2.8836
	NN	1.9880	3.3949	4.1616	8.2320
	NN + PCA	1.1317	2.1448	3.5142	3.7209
	GPR	0.051669	0.17366	0.21046	0.22356

## Data Availability

The data presented in this study are not publicly available due to confidentiality obligations and non-disclosure agreements (NDAs) with the industrial partner involved in this research.

## References

[1] Adhanom I.B., MacNeilage P., Folmer E. (2023). Eye Tracking in Virtual Reality: A Broad Review of Applications and Challenges. Virtual Real..

[2] Stuart S. (2022). Eye Tracking: Background, Methods, and Applications.

[3] Cazzato D., Evangelista A., Leo M., Carcagnì P., Distante C. (2016). A low-cost and calibration-free gaze estimator for soft biometrics: An explorative study. Pattern Recognit. Lett..

[4] Leo M., Cazzato D., Marco T.D., Distante C. (2013). Unsupervised approach for the accurate localization of the pupils in near-frontal facial images. J. Electron. Imaging.

[5] D’Orazio T., Leo M., Distante A. (2004). Eye detection in face images for a driver vigilance system. Proceedings of the IEEE Intelligent Vehicles Symposium.

[6] Torok N., Victor Guillemin J., Barnothy J.M. (1951). Photoelectric Nystagmography. Ann. Otol. Rhinol. Laryngol..

[7] Russo J.E. (1975). The limbus reflection method for measuring eye position. Behav. Res. Methods Instrum..

[8] Wheeless L.L., Boynton R.M., Cohen G.H. (1966). Eye-Movement Responses to Step and Pulse-Step Stimuli. J. Opt. Soc. Am..

[9] Pezzoli C., Santoro E., Paracchini M.B.M., Marano G., Bani D., Raduzzi L.F., Crafa D.M., Carminati M., Merigo L., Ongarello T. (2025). Low-Power Hierarchical Network: Pervasive Eye-Tracking on Smart Eyewear. Proceedings of the 2025 Symposium on Eye Tracking Research and Applications.

[10] Pettenella A., Crafa D.M., Spagnoli J., Pezzoli C., Paracchini M., Di Giacomo S., Fiorini C., Byrne S., Ongarello T., Merigo L. (2025). Development of a Low-Power Wearable Eye Tracker based on Hidden Photodetectors. Proceedings of the 2025 Symposium on Eye Tracking Research and Applications.

[11] Li T., Liu Q., Zhou X. (2017). Ultra-Low Power Gaze Tracking for Virtual Reality. Proceedings of the 15th ACM Conference on Embedded Network Sensor Systems.

[12] Rigas I., Raffle H., Komogortsev O. (2017). Photosensor Oculography: Survey and Parametric Analysis of Designs Using Model-Based Simulation. IEEE Trans.-Hum.-Mach. Syst..

[13] Angelopoulos A.N., Martel J.N., Kohli A.P., Conradt J., Wetzstein G. (2021). Event-Based Near-Eye Gaze Tracking Beyond 10,000 Hz. IEEE Trans. Vis. Comput. Graph..

[14] Rigas I., Raffle H., Komogortsev O.V. (2017). Hybrid PS-V Technique: A Novel Sensor Fusion Approach for Fast Mobile Eye-Tracking with Sensor-Shift Aware Correction. IEEE Sens. J..

[15] Palmero C., Komogortsev O.V., Escalera S., Talathi S.S. (2023). Multi-Rate Sensor Fusion for Unconstrained Near-Eye Gaze Estimation. Proceedings of the 2023 Symposium on Eye Tracking Research and Applications.

[16] Krejtz K., Duchowski A.T., Niedzielska A., Biele C., Krejtz I. (2018). Eye tracking cognitive load using pupil diameter and microsaccades with fixed gaze. PLoS ONE.

[17] Duchowski A.T., Krejtz K., Zurawska J., House D.H. (2020). Using Microsaccades to Estimate Task Difficulty During Visual Search of Layered Surfaces. IEEE Trans. Vis. Comput. Graph..

[18] Crafa D.M., Polonelli T., Pezzoli C., Carminati M., Magno M. (2025). A Low-Power, Non-Invasive and Contactless Eye Blink Detection Sensor Enabling Human-Machine Interfaces for Smart Eyewear Applications. Proceedings of the 2025 Symposium on Eye Tracking Research and Applications.

[19] Fischer R. (2008). Optical System Design.

[20] Smith W. (2007). Modern Optical Engineering.

[21] Druzhin V.V., Tsapenko A.P. (2010). Optical system of human eye model. Proceedings of the IONS 8.

[22] Vogel A., Dlugos C., Nuffer R., Birngruber R. (1991). Optical properties of human sclera, and their consequences for transsclera laser applications. Lasers Surg. Med..

[23] Conrady A. (2013). Applied Optics and Optical Design, Part One.

[24] Zhang Z., Yi D., Lei Z., Li S.Z. (2011). Face liveness detection by learning multispectral reflectance distributions. Proceedings of the 2011 IEEE International Conference on Automatic Face & Gesture Recognition.

[25] Cohen M., Wallace J. (1993). Radiosity and Realistic Image Synthesis.

[26] Koppal S.J. (2014). Lambertian Reflectance. Computer Vision.

[27] Pharr M., Jakob W. (2023). Physically Based Rendering.

[28] (2023). MATLAB.

[29] Wang D., Mulvey F.B., Pelz J.B., Holmqvist K. (2017). A study of artificial eyes for the measurement of precision in eye-trackers. Behav. Res. Methods.

[30] Wu R.J., Clark A.M., Cox M.A., Intoy J., Jolly P.C., Zhao Z., Rucci M. (2023). High-resolution eye-tracking via digital imaging of Purkinje reflections. J. Vis..

[31] Turin G. (1960). An introduction to matched filters. IRE Trans. Inf. Theory.

[32] Wang J. (2023). An Intuitive Tutorial to Gaussian Process Regression. Comput. Sci. Eng..

